# Prevalence of nutritional anemia and its risk factors in children under five in the Gaza Strip

**DOI:** 10.3389/fnut.2025.1496494

**Published:** 2025-02-12

**Authors:** Abdel Hamid El Bilbeisi

**Affiliations:** ^1^Department of Clinical Nutrition, Faculty of Pharmacy, Al Azhar University of Gaza, Gaza, Palestine; ^2^Department of Nutrition, School of Medicine and Health Sciences, University of Palestine, Gaza, Palestine

**Keywords:** Gaza, iron deficiency anemia, nutrition, prevalence, risk factors, under five children

## Abstract

**Background:**

The present study aimed to determine the prevalence of anemia and its associated risk factors among under-five children aged 24–59 months in Gaza Strip, Palestine.

**Method:**

This cross-sectional study was conducted in 2023 before Gaza war, among a representative sample of under-five children. A total of 750 children, aged 24–59 months, were selected from all Gaza Strip governorates, using a cluster random sampling method. Children and their parents were assessed using an interview-based questionnaire, anthropometric measurements, biochemical measurements, and clinical examination. Statistical analysis was performed using SPSS version 25.

**Results:**

The prevalence of anemia (Hb <11 g/dL) among under-five children was 35.6%. Of them, 77.5% had mild anemia and 22.5% had moderate anemia. The prevalence of iron deficiency anemia (IDA) (Hb <11 g/dL and SF <12 μg/L) among under-five children was 4.0%. Various risk factors were found to be statistically significant in relation to nutritional anemia. These included child-related factors such as age, weight, height, sibling arrangement, governorate, and place of residence. Family characteristics like the father’s age, smoking habits, the mother’s education, employment, marital history, income, housing ownership, and a history of anemia during pregnancy also played a role. Additionally, dietary factors such as tea, meat, crisps, cola, fruits, breastfeeding practices, malnutrition, appetite, and hereditary diseases were significantly associated with anemia in these children (*p*-values <0.05 for all).

**Conclusion:**

Our study clearly demonstrates that anemia is highly prevalent (35.6% anemia, and 4.0% IDA) among under-five children in Gaza Strip governorates. Different risk factors including the child’s and family characteristics, and the consumption of some of food items were associated with a high prevalence of anemia. The high prevalence of anemia should be seriously considered and policy makers should take steps to reduce it.

## Introduction

Anemia has been recognized as a significant global public health issue, mostly impacting young children, adolescent girls, women of reproductive age (WRA), pregnant and postpartum women (1). Anemia is a deficiency in the size or number of red blood cells (RBCs), or a lower-than-normal level of hemoglobin (Hb) within the RBCs (2). The pathophysiology of anemia involves various mechanisms depending on its underlying cause. These mechanisms can be grouped into three main categories: impaired red blood cell production, increased red blood cell destruction, and blood loss (1, 2). In 2019, the World Health Organization (WHO) estimated that approximately 40% of under-five children, 37% of pregnant women, and 30% of WRA worldwide are anemic (3).

Nutritional anemia is a result of inadequate nutritional needs to meet the requirements for the production of Hb and RBCs (4). Iron is a vital element in Hb; however, other micronutrients like vitamin A, vitamin B2, vitamin B6, vitamin B9, vitamin B12, vitamins C, D, and E, as well as minerals like copper and zinc are also essential for Hb formation or play distinct roles in iron absorption and immune function (5). Their deficiency or impaired absorption can potentially increase the risk of anemia (6). The most prevalent cause of anemia globally is believed to be iron deficiency, present in around 25 to 50% of children under-five and WRA with anemia, although other nutritional deficiencies also contribute to anemia (7). Symptoms of anemia vary depending on its severity. However, common symptoms include fatigue, dizziness, shortness of breath, chills at the extremities, headache, palpitations, decreased physical endurance, inadequate nutrition, and reduced productivity (8).

In children, anemia can increase susceptibility to infections and have adverse effects on physical growth, motor and cognitive development, productivity and school performance, thus impairing their growth, while also increasing the risk of other health-related problems (9). Anemia and iron deficiency anemia (IDA) remain a high burden and a malnutrition challenge in the Eastern Mediterranean Region, particularly among children and women (3).

Among under-five children, the prevalence of anemia in the Eastern Mediterranean Region ranged between 11.9 and 79.5%, whereby the lowest estimate was reported in Jordan and the highest in Yemen (10). The importance of studying anemia in children under five in the Gaza Strip lies in the fact that there are no previous studies focusing on this age group in the region (3, 10). Anemia represents a significant public health issue that greatly affects children’s physical and mental growth and development. Understanding the factors contributing to anemia is crucial for improving children’s health and reducing associated risks. Through this study, the main causes of anemia in the Gaza Strip can be highlighted, which will help design preventive and therapeutic strategies aimed at improving the health of children in this age group and alleviating the health and economic burdens on the community. Therefore, the current study aimed to determine the prevalence of anemia and its associated risk factors among under-five children aged 24–59 months in Gaza Strip, Palestine.

## Materials and methods

### Study design

This observational, analytical, cross-sectional study was conducted among a representative sample of under-five children aged 24–59 months in the Gaza Strip governorates.

### Study location and period

The current study was conducted in 2023 before Gaza war, in the households of the Gaza Strip, Palestine. The estimated population of the Gaza Strip is about 2,106,745 million (11). The Gaza Strip is divided into five governorates: North Gaza, Gaza City, Middle-Area, Khan Younis, and Rafah (12).

### Study population

A total of 750 children and their proxy were selected from all Gaza Strip governorates, using a cluster random sampling method. Households having at least one child (male or female), aged 24 to 59 months, and living with his/her mother in the same household, and mothers and fathers aged ≥18 years and having under-five children aged 24 to 59 months were included in the present study. On the contrary, households without under-five children, under-five children with disabilities or chronic disease, preterm infants (<37 weeks), infants of diabetic mothers, and under-five children who have a history of complications during delivery (aspiration or trauma) were excluded from the present study.

### Sample size and sampling technique

The current study used the Charan and Biswas formula (13) to obtain a representative sample size of 680 under-five children, both gender, aged 24 to 59 months, and to which we added 10% as an expected non-response rate. Finally, a total of 750 under-five children and their proxy were responded and recruited. The sample was proportionally selected from the five governorates of the Gaza Strip, using a cluster random sampling method based on the population density in each of them as follows: 240 children from North Gaza, 219 from Gaza City, 150 from the Middle Area, 105 from Khan Younis, and 36 from Rafah governorate.

### Data collection

#### Interview-based questionnaire

A pretested structured questionnaire was used, the questionnaire was consisting of demographic socio-economic characteristics of the children and their families; children health and nutrition characteristics including feeding practices, food consumption pattern and the health condition. Ten trained interviewers conducted the poll in all five governorates at the same time.

#### Anthropometric measurements

The height (cm) and weight (kg) of children were measured following standard recommended procedures. A digital weighing scale (to the nearest 0.1 kg) (SECA, Germany) and a body meter (with the precision of 0.1 cm) (SECA, Germany) were used. The measurement for each child was carried out twice, and the average reading was documented as the final reading (14).

#### Biochemical measurements

After 12 h fasting, a venous blood samples were collected from the children participating in the study at their homes by five qualified nurses trained to handle children during the blood collection process. The blood collection was carried out with the help of the children’s mothers or fathers to ensure the children’s cooperation. To avoid pre-analytical errors, several steps were taken to ensure the accuracy and safety of the samples including: selection of qualified nurses, use of sterile equipment, proper sample storage procedures, timely sending of samples, and accurate documentation. Venous blood (4.0 mL) was drawn into two vacutainer tubes and was used for blood analysis. One mL of blood was used for Hb test (mg/dL), and the remaining 3.0 mL of blood was used for serum ferritin (SF) test (μg/L). In the current study, the cyanmethemoglobin method was used for measuring of Hb level using a hemoglobinometer device; in addition, Mindray BS-300 chemistry analyzer instrument was used to measure SF using ARCHITECT Ferritin 7K59 kit (15). Furthermore, Hb test was measured for all children, and if the children had anemia (Hb <11 mg/dL), the SF test was conducted. The laboratory tests were analyzed in a private licensed laboratory.

In the current study, anemia was diagnosed, and categorized as mild, moderate, or severe by assessing the levels of Hb in the blood and based on the WHO specific cut-offs for under-five children aged 24 to 59 months as follows: mild (Hb = 10.0–10.9 g/dL), moderate (Hb = 7.0–9.9 g/dL), severe (Hb <7.0 g/dL), and normal (Hb ≥11.0 g/dL). Additionally, IDA is defined according to Hb and SF levels, as follows: Hb <110 g/L and SF <12 μg/L for under-five children (10).

#### Clinical examination

The study participants were examined by the physicians for signs and symptoms of anemia which includes: pallor, fatigue, dizziness, shortness of breath, chills at the extremities, headache, palpitations, decreased physical endurance, inadequate nutrition, and reduced productivity (8).

#### Pilot study

A pilot study was conducted with a sample of 20 participants to test the questionnaire and data collection procedures. Based on the feedback from this pilot, necessary adjustments were made to the questionnaire to ensure clarity and accuracy in the main study.

#### Statistical analysis

Statistical analysis was performed using SPSS version 25. Data are expressed as means ± SD for continuous variables and as percentage for categorical variables. The differences between means were tested by using independent sample *t*-test. The chi-square test was used to examine differences in the prevalence of different categorical variable. *p*-value less than 0.05 was considered as statistically significant.

## Results

A total of 750 under-five children aged 24 to 59 months were included in the final analysis. Of them, 43.6% were males and 56.4% were females. The mean age of the study participants was 3.58 ± 0.61 years. The findings demonstrated that 65.2% of children aged four to less than 5 years old. The mean weight of children at birth was 3,187 ± 416.3 grams, the child’s current weight (kg) was 16.81 ± 3.08, and the child’s current height (cm) was 108.53 ± 8.59. The mean of the child’s arrangement between his brothers and sisters was 2.11 ± 1.09. In addition, 4.8% of the participants were from Rafah, 14.0% were from Khan Yunis, 20.0% were from the Middle Area, 29.2% were from Gaza City, and 32.0 were from North Gaza. Regarding the place of residence, the results revealed that 49.2, 15.2, and 35.6% were residents in city, village, and camp, respectively. A large percentage (78.8%) of the study participants were refugees, and only 21.2% were citizens as shown in Table 1.

**Table 1:** The characteristics of the study participants (under-five children).

Variables	*N* = 750	100%
Gender		
Males	327	43.6
Females	423	56.4
The age of the child (years), mean ± SD: 3.58 ± 0.61		
Two to less than three years	51	6.8
Three to less than four years	210	28.0
Four to less than five years	489	65.2
Child weight at birth (grams)		
Mean ± SD	3,187 ± 416.3	
The child’s current weight (kg)		
Mean ± SD	16.81 ± 3.08	
Child’s current height (cm)		
Mean ± SD	108.53 ± 8.59	
The child’s arrangement between his brothers and sisters		
Mean ± SD	2.11 ± 1.09	
Governorate		
Rafah	36	4.8
Khan Yunis	105	14.0
Middle Area	150	20.0
Gaza	219	29.2
North Gaza	240	32.0
Place of residence		
City	369	49.2
Village	114	15.2
Camp	267	35.6
Citizenship		
Refugee	591	78.8
Citizen	159	21.2
The child’s blood hemoglobin concentration (mg/dL)		
Mean ± SD	11.21 ± 0.89	
Hemoglobin <11 mg/dL (anemia)	267	35.6
Hemoglobin 11 mg/dL or more (no anemia)	483	64.4
Hemoglobin <11 g/dL and serum ferritin <12 μg/L		
Iron deficiency anemia	30	4.0
No iron deficiency anemia	720	96.0
The following laboratory tests only for the child’s with anemia (*n* = 267)		
Mild anemia		
Hemoglobin 10.0–10.9 g/dL	207	77.5
Moderate anemia		
Hemoglobin 7.0–9.9 g/dL	60	22.5
Severe anemia		
Hemoglobin <7.0 g/dL	0.0	0.0

Data are expressed as means ± SD for continuous variables and as percentage for categorical variables.

In addition, the findings revealed that the prevalence of anemia (Hb <11 g/dL) among under-five children in Gaza governorates was 35.6%. Of them, 77.5% had mild anemia and 22.5% had moderate anemia. Furthermore, the prevalence of iron deficiency anemia (Hb <11 g/dL and SF <12 μg/L) among under-five children in Gaza governorates was 4.0% (Table 1).

**Figure 1:**
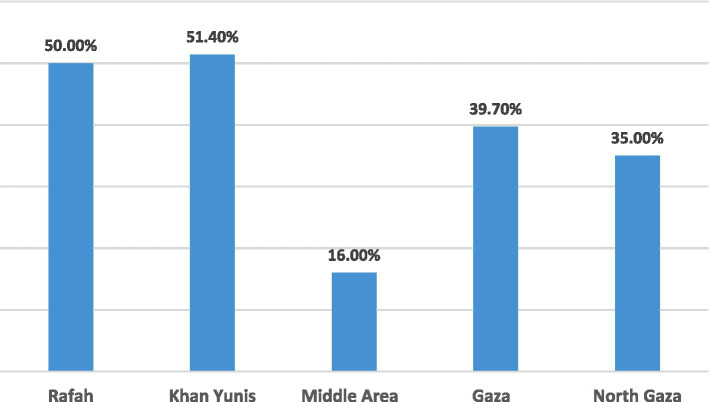
The prevalence of anemia among under-five children aged 24 to 59 months by Gaza governorates.

The prevalence of anemia (Hb <11 g/dL) among under-five children aged 24 to 59 months by Gaza Strip governorates is shown in Figure 1. The findings revealed that the highest prevalence of anemia was in the Khan Yunis governorate (51.4%), while the lowest prevalence of anemia was in the Middle Are governorate (16.0%). In addition, the prevalence of anemia among under-five children aged 24 to 59 months by Gaza governorates was distributed as follows 50.0, 51.4, 16.0, 39.7, and 35.0% in Rafah, Khan Yunis, Middle Area, Gaza city, and North Gaza, respectively.

Relationship between the characteristics of under-five children with nutritional anemia in Gaza governorates is shown in Table 2. The findings demonstrated that for the following variables (age of the children, child weight at birth, the child’s current weight, current height, the child’s arrangement between his brothers and sisters, governorates, and place of residence) statistically significant associations were found between under-five children with and without anemia (*p*-values <0.005 for all).

**Table 2:** Relationship between the characteristics of under-five children with nutritional anemia in Gaza governorates.

Variables	Children with anemia 267 (35.6)	Children without anemia 483 (64.4)	*p*-value
Gender			
Males	114 (42.7)	213 (44.1)	0.468
Females	153 (57.3)	270 (55.9)	
The age of the child (years)			
Mean ± SD	3.41 ± 0.75	3.67 ± 0.50	0.001
Two to < three years	42 (15.7)	9.0 (1.9)	
Three to < four years	72 (27.0)	138 (28.6)	
Four to < five years	153 (57.3)	336 (69.6)	
Child weight at birth (grams)			
Mean ± SD	3020.2 ± 405.9	32,795 ± 393.7	0.001
The child’s current weight (kg)			
Mean ± SD	15.68 ± 3.39	17.44 ± 2.71	0.001
Child’s current height (cm)			
Mean ± SD	104.04 ± 8.32	111.01 ± 7.71	0.001
The child’s arrangement between his brothers and sisters			
Mean ± SD	2.30 ± 1.15	2.00 ± 1.05	0.040
Governorate			
Rafah	18 (6.7)	18 (3.7)	0.008
Khan Yunis	54 (20.2)	51 (10.6)	
Middle Area	24 (9.0)	126 (26.1)	
Gaza	87 (32.6)	132 (27.3)	
North Gaza	84 (31.5)	156 (32.3)	
Place of residence			
City	171 (64.0)	198 (41.0)	0.002
Village	27 (10.1)	87 (18.0)	
Camp	69 (25.8)	198 (41.0)	
Citizenship			
Refugee	219 (82.0)	372 (77.0)	0.226
Citizen	48 (18.0)	111 (23.0)	

Data are expressed as means ± SD for continuous variables and as percentage for categorical variables. The differences between means were tested by using independent sample *t*-test. The chi-square test was used to examine differences in the prevalence of different categorical variable. *p*-value less than 0.05 was considered as statistically significant. SD, standard deviation.

Relationship between the characteristics of the child’s family with nutritional anemia in Gaza governorates is shown in Table 3. The findings demonstrated that for the following variables (father’s age, smoker father, mother’s educational level, mother’s work, number of years of marriage, mother’s history of miscarriages, monthly income of the family, enough income, housing ownership, mothers history of anemia during pregnancy, delivery date, and types of delivery) statistically significant associations were found between under-five children with and without anemia (*p*-values <0.005 for all).

**Table 3:** Relationship between the characteristics of the child’s family with nutritional anemia in Gaza governorates.

Variables	Children with anemia 267 (35.6)	Children without anemia 483 (64.4)	*p*-value
Father’s age (years)			
Mean ± SD	37.02 ± 6.26	34.48 ± 4.86	0.001
Father’s educational level			
Primary	3.0 (1.1)	9.0 (1.9)	0.775
Preparatory	9.0 (3.4)	30 (6.2)	
Secondary	51 (19.1)	72 (14.9)	
Diploma	36 (13.5)	72 (14.9)	
University	168 (62.9)	300 (62.1)	
Is the father work?			
Yes	246 (92.1)	432 (89.4)	0.325
No	21 (7.9)	51 (10.6)	
Is the father a smoker?			
Yes	186 (69.7)	258 (53.4)	0.009
No	81 (30.3)	225 (46.6)	
Mother’s age (years)			
Mean ± SD	32.20 ± 7.16	30.83 ± 4.67	0.069
Mother’s educational level			
Primary	3.0 (1.1)	15 (2.5)	0.011
Preparatory	0.0 (0.0)	0.0 (0.0)	
Secondary	102 (38.2)	105 (21.7)	
Diploma	21 (7.9)	93 (19.3)	
University	141 (52.8)	273 (56.5)	
Is the mother work?			
Yes	36 (13.5)	120 (24.8)	0.023
No	231 (86.5)	363 (75.2)	
Is there a relationship between the father and the mother?			
Yes	54 (20.2)	138 (28.6)	0.096
No	213 (79.8)	345 (71.4)	
If the answer is yes, what is the degree of kinship?			
Cousin	54 (20.2)	138 (28.6)	0.096
No relationship	213 (79.8)	345 (71.4)	
Number of years of marriage			
Mean ± SD	9.37 ± 4.12	8.27 ± 3.56	0.029
Number of children			
Mean ± SD	3.06 ± 1.24	3.20 ± 1.48	0.458
Has the mother suffered from previous miscarriages?			
Yes	108 (40.4)	102 (21.1)	0.001
No	159 (59.6)	381 (78.9)	
Is the mother a smoker?			
Yes	0.0 (0.0)	12 (2.5)	0.170
No	267 (100)	471 (97.5)	
The monthly income of the family (Shekels)			
Mean ± SD	1589.8 ± 840.0	1871.4 ± 1312.5	0.040
<2,000 Shekels	174 (65.2)	285 (59.0)	
≥2,000 Shekels	93 (34.8)	198 (41.0)	
Enough income			
Sufficient for needs with savings	0.0 (0.0)	15 (3.1)	0.001
Just enough	12 (4.5)	138 (28.6)	
Not enough	255 (95.5)	330 (86.3)	
Housing ownership			
Rent	66 (24.7)	69 (14.3)	0.031
Ownership	201 (75.3)	414 (85.7)	
Number of family members inside the house			
Mean ± SD	5.12 ± 1.31	5.16 ± 1.14	0.812
Did the mother suffer from anemia during pregnancy?			
Yes	195 (73.0)	150 (31.1)	0.001
No	72 (27.0)	333 (68.9)	
Did the mother receive iron or vitamins during pregnancy?			
Yes	186 (69.7)	369 (76.4)	0.156
No	81 (30.3)	114 (23.6)	
Delivery date			
Completed	222 (83.1)	462 (95.7)	0.001
Early	45 (16.9)	21 (4.3)	
Type of delivery			
Normal	162 (60.7)	363 (75.2)	0.013
Cesarean section	105 (39.3)	120 (24.8)	

Data are expressed as means ± SD for continuous variables and as percentage for categorical variables. The differences between means were tested by using independent sample *t*-test. The chi-square test was used to examine differences in the prevalence of different categorical variable. *p*-value less than 0.05 was considered as statistically significant. SD, standard deviation.

Relationship between the characteristic of the child health and nutrition with nutritional anemia in Gaza governorates is shown in Table 4. The findings demonstrated that for the following variables (child drinks tea, times of tea drinking per day, drinking of tea immediately after eating or with food, number of meals per day, times of meat (calf/birds/fish/liver) eating per week, times of crisps (chips and their derivatives) eating per week, times of cola and soft drinks drinking per week, eat of fruits regularly, type of breastfeeding, suffering from malnutrition, child’s appetite, hereditary diseases in the family/family, and types of hereditary diseases) statistically significant associations were found between under-five children with and without anemia (*p*-values <0.005 for all).

**Table 4:** Relationship between the characteristic of the child health and nutrition with nutritional anemia in Gaza governorates.

Variables	Children with anemia 267 (35.6)	Children without anemia 483 (64.4)	*p*-value
Does the child drink tea?			
Yes	74 (83.1)	61 (37.9)	0.001
No	15 (16.9)	100 (62.1)	
If the answer is yes, how many times a day?			
One time	56 (62.9)	45 (28.0)	0.001
Two times	18 (20.2)	14 (8.7)	
Three times	0.0 (0.0)	2.0 (1.2)	
Not drink tea	15 (16.9)	100 (62.1)	
Does the child drink tea immediately after eating or with food?			
Yes	58 (65.2)	46 (28.6)	0.001
No	31 (34.8)	115 (71.4)	
How many meals does the child eat daily?			
One meal	0.0 (0.0)	4.0 (2.5)	0.001
Two meals	49 (55.1)	48 (29.8)	
Three meals	36 (40.4)	100 (62.1)	
Four meals	4.0 (4.5)	9.0 (5.6)	
How many times does a child eat meat (calf/birds/fish/liver) per week?			
Mean ± SD	1.28 ± 0.69	2.02 ± 1.30	0.001
How many times does a child eat crisps (chips and their derivatives) per week?			
Mean ± SD	5.13 ± 2.39	3.61 ± 2.59	0.001
How many times does a child drink cola and soft drinks per week?			
Mean ± SD	3.52 ± 2.81	2.08 ± 2.48	0.001
Does the child drink ready-made juices?			
Yes	55 (61.8)	89 (55.3)	0.194
No	34 (38.2)	72 (44.7)	
Does the child eat fruits regularly?			
Yes	15 (16.9)	90 (55.9)	0.001
No	74 (83.1)	71 (44.1)	
Type of breastfeeding			
Natural	59 (66.3)	115 (7.4)	0.029
Artificial milk	17 (19.1)	13 (8.1)	
Both types	13 (14.6)	33 (20.5)	
Did the child suffer or is suffering from malnutrition?			
Yes	56 (62.9)	21 (13.0)	0.001
No	33 (37.1)	140 (87.0)	
Did the child suffer from parasitic worms?			
Yes	20 (22.5)	32 (19.9)	0.371
No	69 (77.5)	129 (80.1)	
Has the child suffered or is suffering from recurrent or chronic diarrhea?			
Yes	6.0 (6.7)	15 (9.3)	0.327
No	83 (93.3)	146 (90.7)	
Has the child received or is he receiving iron or vitamins during the past two months?			
Yes	2.0 (2.2)	0.0 (0.0)	0.126
No	87 (97.8)	161 (100)	
How you evaluate the child’s appetite?			
Good	2.0 (2.2)	72 (44.7)	0.001
Acceptable	58 (65.2)	71 (44.1)	
Poor	29 (32.6)	18 (11.2)	
Does the child suffer from any diseases?			
No	89 (100)	161 (100)	—
Are there hereditary diseases in the family/family?			
Yes	22 (24.7)	17 (10.6)	0.003
No	67 (75.3)	144 (89.4)	
If the answer is yes, mention it			
Hypertension	9.0 (10.1)	12 (7.5)	0.002
Diabetes mellitus	7.0 (7.9)	5.0 (3.1)	
CVDs	6.0 (6.7)	0.0 (0.0)	
No hereditary diseases	67 (75.3)	144 (89.4)	

Data are expressed as means ± SD for continuous variables and as percentage for categorical variables. The differences between means were tested by using independent sample *t*-test. The chi-square test was used to examine differences in the prevalence of different categorical variable. *p*-value less than 0.05 was considered as statistically significant. SD, standard deviation; CVDs, cardiovascular diseases.

## Discussion

Anemia remains a significant public health concern, with the most vulnerable being under-five children, adolescent girls, WRA and pregnant women (1). Anemia mainly affects low-income and middle-income countries with the heaviest burden, specifically populations residing in rural areas, economically disadvantaged households, and those lacking formal education (3). Globally, it was estimated that 40% of under-five children are affected by anemia (16). To the best of our knowledge no recent data about the prevalence of anemia among under-five children in Palestine and in the Gaza Strip. In addition, national studies on IDA in the EMR countries are quite limited (3, 10). Therefore, the current study aimed to determine the prevalence of anemia and its associated risk factors among under-five children aged 24–59 months in Gaza Strip, Palestine.

The main findings of the current study revealed that anemia is highly prevalent (35.6%) among under-five children aged 24 to 59 months in Gaza Strip governorates. In addition, the highest prevalence of anemia was in Khan Yunis governorate, while the lows prevalence was in Middle Area governorate. In the EMR, the prevalence of anemia ranged between 11.9 and 79.5% among under-five children, whereby the lowest estimate was reported in Jordan and the highest in Yemen (10). In 2019, the WHO estimated that approximately 40% of under-five children are anemic (3). This difference between countries in the prevalence of anemia among under-five children, could be attributed to the variation in the characteristics of the study population, as well as variation in the demographic socio-economic status, as it is documented that the prevalence of anemia increases with increasing poverty and lacking formal education (17). Furthermore, our results are comparable to the results of a previous study, which reported significantly different prevalence’s of anemic children between the different regions of the Gaza Strip, Palestine (18).

The findings showed that among the anemic children, 77.5% had mild anemia and 22.5% had moderate anemia. The WHO in 2024, reported that the majority of EMR countries fall into the moderate category for anemia. Pakistan, Somalia and Yemen fall into the severe category for all three age/population groups that were considered, i.e., under five children, WRA and pregnant women. As for the mild category, Jordan and Kuwait have reported mild anemia prevalence’s among under five children, while only Jordan reported mild anemia among pregnant women (3). The results of the current study showed that the majority (77.5%) of anemic under-five children had mild anemia.

In the current study, the prevalence of IDA among under-five children aged 24 to 59 months in Gaza governorates was 4.0%. National studies on IDA in the EMR are quite limited; in general, the prevalence of IDA was lower among under five children as compared to WRA, with the exception of Iraq and Pakistan. As for under five children, the range was between 2.9% in Oman, 5.1% in Jordan and 28.6% in Pakistan (3).

On the other hand, the findings demonstrated that different risk factors including the child’s and family characteristics, and the consumption of some of food items were associated with a high prevalence of anemia. Previous studies conducted in low-income countries showed that young age was a significant independent risk factor associated with anemia, and children with low age were twice as likely to have anemia compared to children with high age (19, 20). Baranwal et al. (21) showed that there was no impact of place of residence on anemia. A previous study showed that anemic children had significantly lower body weight, height and weight for age (22). The results of the current study support these findings.

A previous study in Gaza Strip showed that low education level of the parents and smoking are significant risk factors for anemia in children (23). The results of the current study support these findings. In addition, increased years of marriage could increase the number of family members and due to high poverty level in the Gaza Strip all could increase the risk of household’s food insecurity and risk of anemia. Furthermore, a previous study showed that mothers with spontaneous abortion could increase the risk of anemia in children aged 3–7 years old (24). Andargie et al. (25) showed that low family income and poverty were significantly associated with anemia, which resulted in insufficient nutrition and inadequate health care as well as educated states. Endris et al. (26) showed that the prevalence of anemia was higher among children of anemic mothers. Moreover, Li et al. (27) showed that cesarean delivery is associated with increased anemia in children aged 12 and 58 months. The results of the current study support these findings. Our findings support those from other studies that reported reduced placenta-to-fetus cord blood transfusion and decreased iron storage at birth in cesarean-delivered infants (28, 29). Additionally, inappropriate dietary choices and frequently consumption of tea, chips and their derivatives and cola with meals are associated risk factors for anemia (30). Our results also, agrees with previous studies reporting that the intake of tea was significantly high among anemic subjects (31, 32). In addition, the results of the current study showed that children who consumed fruits regularly were at low risk of anemia as these foods contains micronutrients which could decrease the risk of anemia among under-five children. Moreover, another study reported that cola consumption significantly increased the risk of anemia (33). The harmful effect of high caffeine beverages (tea, coffee, and cocoa) on anemia may be justified as they contain polyphenols (tannins) that inhibit absorption of iron from intestine (32). Moreover, breastfeeding remains overwhelmingly beneficial for a child’s development (34). Anemia in under-five children is most often attributed to iron deficiency; other nutritional, infectious, and hereditary factors could contribute to high risk of anemia (35). The results of the current study support these findings. Additionally, dietary inhibitors of iron absorption including whole grains due to the presence of bran, polyphenols, phytate, and calcium. However, because calcium is an essential nutrient, it cannot be considered as an inhibitor of iron absorption in the same way as phytates or polyphenols. To minimize this interaction, certain practices should be adopted, such as increasing iron intake and its bioavailability, or avoiding the consumption of calcium-rich foods and iron-rich foods in the same meal. When these foods are consumed, they should be consumed 1–2 h after having the iron-rich meal or snack (3). Moreover, these inhibitors were shown to have a notable effect on non-heme iron mainly (present in plant food sources), whereas heme iron (present in animal food sources) is affected to a lesser extent (3, 32).

Finally, given the complex etiology of anemia, successful and effective anemia reduction efforts should not focus solely on iron, but rather should identify all additional contributing nutritional and social factors in order to develop and implement an evidence-based set of interventions, tailored to the context and to the locally identified determinants.

### Strengths and limitations

The main strength of our study was its being one of the first studies, which shows the prevalence of anemia and its associated risk factors among under-five children aged 24–59 months in Gaza Strip, Palestine, and its large, representative sample size. On contrary, this study shares the standard limitation of cross-sectional design, challenging to make a causal association. Besides, information was collected from household heads (mothers/fathers) it was likely to have recall bias.

## Conclusion

Our study clearly demonstrates that anemia is highly prevalent (35.6%) among under-five children aged 24 to 59 months in Gaza Strip governorates. Of them, 77.5% had mild anemia and 22.5% had moderate anemia. The prevalence of IDA among under-five children aged 24 to 59 months in Gaza governorates was 4.0%. The prevalence of anemia (Hb <11 g/dL) by Gaza governorates was distributed as follows 50.0, 51.4, 16.0, 39.7, and 35.0% in Rafah, Khan Yunis, Middle Area, Gaza city, and North Gaza, respectively. In addition, different risk factors including the child’s and family characteristics, and the consumption of some of food items were associated with a high prevalence of anemia. The high prevalence of anemia among under-five children aged 24 to 59 months in the Gaza Strip governorates should be seriously considered and policy makers should take steps to reduce it.

## Data Availability

The raw data supporting the conclusions of this article will be made available by the authors, without undue reservation.
